# Editorial

**DOI:** 10.1093/rpd/ncaf085

**Published:** 2025-08-28

**Authors:** Eva Forssell-Aronsson, Christian Bernhardsson

**Affiliations:** Department of Medical Radiation Sciences, Institute of Clinical Sciences, Sahlgrenska Academy, University of Gothenburg, SE 413 45 Gothenburg, Sweden; Department of Medical Physics and Biomedical Engineering, Sahlgrenska University Hospital, SE 413 45 Gothenburg, Sweden; Medical Radiation Physics, Department of Translational Medicine, Lund University, SE 205 02 Malmö, Sweden

This issue of *Radiation Protection Dosimetry* is based on contributions to the XIX conference of the Nordic Society for Radiation Protection (NSFS), held at Malmö Live, Malmö, Sweden, from 5 to 9 June 2023.

NSFS was founded in 1964 at the initiative of Rolf Sievert and includes members from all five Nordic countries. The task of NSFS is to activate the exchange of knowledge and experience in the Nordic countries regarding protection against ionizing and non-ionizing radiation for all kinds of occupational, medical, or public exposures. NSFS was a founding member of the International Radiation Protection Association (IRPA). Since 1966, NSFS has held regular conferences at three- or four-year intervals, in turn in each of the Nordic countries. In addition, NSFS has arranged various meetings on selected important themes.

The theme of the XIX conference was ‘Sharing and Caring’, which can be interpreted as sharing of knowledge and caring about negative effects of radiation and its benefits for individuals and society. The conference started with excursions to the European Spallation Source (ESS) in Lund, Lund University Bioimaging Centre (LBIC), historical sites in Lund, and the Emergency Preparedness Laboratory in Malmö. The scientific committee received and evaluated many high-quality abstracts that formed the basis of the program. The conference program consisted of 58 oral presentations and 24 posters on a variety of radiation protection aspects. Since travelling and participation in meetings have decreased after the pandemic, we were pleased that more than 110 participants from 11 countries attended the conference.

The conference started traditionally with the Bo Lindell lecture, which was given by Ritva Bly from the Radiation and Nuclear Safety Authority (STUK) in Helsinki, Finland. The lecture was entitled ‘Trends in collective effective doses from radiological procedures’.

Invited presentations were given by the IRPA President Bernard Le Guen and the International Commission on Radiological Protection (ICRP) Chair Werner Rühm. Jack Valentin and Sören Mattsson presented the history of NSFS, a topic valuable both for present, new, and potential NSFS members. The R/N situation in Ukraine and possible consequences were described and summarized by Vadim Chumak from the National Academy of Medical Sciences of Ukraine in Kyiv. Other themes were *International, European, and Nordic cooperation in radiation protection*, *AI in radiation protection*, *Future competence supply of radiation professionals*, and a special evening session organized by and for young radiation professionals. The remaining oral presentations were given in sessions entitled *Decommissioning, Emergency preparedness, Environmental monitoring, Environmental radiology, European Spallation Source, Medical applications, Radiation dose modeling, Radiobiology, Radioecology*, and *Radon*.

Eva Forssell-Aronsson, President of NSFS 2019–2023

Christian Bernhardsson, Vice President of NSFS 2019–2023

